# Additive and non-additive epigenetic signatures of natural hybridization between fish species with different mating systems

**DOI:** 10.1080/15592294.2022.2123014

**Published:** 2022-09-15

**Authors:** Waldir M. Berbel-Filho, George Pacheco, Mateus G. Lira, Carlos Garcia de Leaniz, Sergio M. Q. Lima, Carlos M. Rodríguez-López, Jia Zhou, Sofia Consuegra

**Affiliations:** a Centre for Sustainable Aquatic Research, Department of Biosciences, College of Science, Swansea University, Swansea, UK; b Section for Evolutionary Genomics, The Globe Institute, Faculty of Health and Medical Sciences, University of Copenhagen, Copenhagen, Denmark; c Laboratório de Ictiologia Sistemática e Evolutiva, Departamento de Botânica e Zoologia, Universidade Federal do Rio Grande, Natal, Brazil; d Environmental Epigenetics and Genetics Group, Department of Horticulture, College of Agriculture, Food and Environment, University of Kentucky, Lexington, KY, USA; e State Key Laboratory of Plant Genomics, Institute of Microbiology, Chinese Academy of Sciences, Beijing, China

**Keywords:** DNA methylation, self-fertilization, outcrossing, epigenetic diversity, mangrove killifish

## Abstract

Hybridization is a major source of evolutionary innovation. In plants, epigenetic mechanisms can help to stabilize hybrid genomes and contribute to reproductive isolation, but the relationship between genetic and epigenetic changes in animal hybrids is unclear. We analysed the relationship between genetic background and methylation patterns in natural hybrids of two genetically divergent fish species with different mating systems, *Kryptolebias hermaphroditus* (self-fertilizing) and *K. ocellatus* (outcrossing). Co-existing parental species displayed highly distinct genetic (SNPs) and methylation patterns (37,000 differentially methylated cytosines). Hybrids had predominantly intermediate methylation patterns (88.5% of the sites) suggesting additive effects, as expected from hybridization between genetically distant species. The large number of differentially methylated cytosines between hybrids and parental species (n = 5,800) suggests that hybridization may play a role in increasing genetic and epigenetic variation. Although most of the observed epigenetic variation was additive and had a strong genetic component, we also found a small percentage of non-additive, potentially stochastic, methylation differences that might act as an evolutionary bet-hedging strategy and increase fitness under environmental instability.

## Introduction

Hybridization is a source of evolutionary novelty [1,2] and can promote speciation through the generation of genetic and phenotypic variation, which could precede adaptive radiation [3,4]. Hybridization can result in heterosis or hybrid vigour, with hybrids displaying higher performance than the parents [5], but also in hybrid incompatibility resulting in loss of fitness, increased mortality, and increased reproductive isolation between parental species [6]. Interspecific hybridization rates are particularly high in plants (40%) [7] and seems to be a phenomenon uniformly distributed across animals [8]. Hybrid incompatibility, encompassing hybrid inviability and sterility is, together with reduced hybrid fitness, one of the strongest postzygotic mechanisms acting as isolating barriers for interspecific hybridization [9]. Hybrid incompatibility can be caused by interactions between incompatible parental genetic alleles [10,11], changes in regulatory elements [12], transposable element activity [13,14] or chromosomal rearrangements [15], and also by cytosine methylation [16,17] and changes in gene expression patterns [18,19]. For instance, a single epigenetically inactivated gene (HISN6B) is responsible for hybrid incompatibility between strains of *Arabidopsis thaliana* [6].

Interactions between parental genomes can result in additive or non-additive gene expression patterns [20]. For example, hybrids between farmed and wild Atlantic salmon or between recently-diverged pupfish species have shown mostly non-additive patterns of gene expression (e.g., over or under-dominance) relatively to the parental species [18,21], while hybrids of house mouse subspecies and *Drosophila* species display predominately additive effects [22,23]. As species diverge, additivity in hybrids becomes more likely compared to patterns of dominance [22], but the epigenetic effects of hybridization are less known, particularly in animals. In some cases like allopolyploid plant hybrids, DNA methylation plays a central role in the initial stabilization of the genomefor example, through gene silencing and dosage compensation [24]. However, it is unclear to what extent the role of epigenetic modifications in the restructuring of the hybrid genomes is determined by the underlying genetic changes [25]. In plant hybrids, both additive [26] and non-additive [27] effects can modify DNA methylation patterns, the direction of which depends on the initial degree of divergence between parental lineages [28]. For example, hybrids between inbred lines of *Arabidopsis* display non-additive changes in DNA methylation more frequently when parental methylation levels are different, but not when they are similar [29]. Hybridization can also lead to demethylation of transposable elements, which can result in hybrid disfunction and potentially postzygotic isolation [14,30]. Thus, epigenetic changes during hybridization are related to changes in gene regulation and potentially to reproductive isolation which could lead to speciation, but the extent to which these changes are dependent on the genetic background and the distance between the parental species is unclear, particularly in animals [25]. In some hybrid fish with clonal reproduction, epigenetic variation contribute to phenotypic plasticity and allows them to cope with environmental uncertainty [31].

In general, organisms that inhabit predictable environments seem to display more environmentally directed epigenetic changes, in contrast to those living in more unpredictable environments for which stochastic epigenetic changes (spontaneous epimutations) are more common [32]. However, the relative dependence of epigenetic variation from the genotype as well as its transgenerational stability seems to be system-dependent [33]. Here, we analysed the epigenetic (DNA methylation) patterns of hybrids between two genetically distant mangrove killifish species with different mating systems (defined here as the relative frequency of cross-fertilization or outcrossing and self-fertilization in a population [34]), *Kryptolebias hermaphroditus* (predominantly self-fertilizing) and *K. ocellatus* (outcrossing) [35]. We compared epigenetic (methylation) and genetic (SNPs) patterns of variation between hybrids and their parental species, to assess the extent to which hybrids’ methylation is determined by the genomic background and whether, as in plants, they mainly display additive patterns.

## Material and methods

### Study species

*Kryptolebias hermaphroditus* and *K. ocellatus* are two mangrove killifish species that coexist in intermittent mangrove microhabitats in southeast Brazil. *Kryptolebias hermaphroditus* is one of the two known examples of self-fertilizing hermaphroditism in vertebrates [36], its populations mainly consisting of selfing hermaphrodites and males at low frequencies [37,38]. Outcrossing rarely occurs between *K. hermaprhoditus* males and hermaphrodites [39] and selfing is the major mode of reproduction [40]. In contrast, *K. ocellatus* populations consist of males and hermaphrodites in approximately equal ratio, and they exclusively reproduce via outcrossing [35]. The differences in mating systems between both species, predominantly selfing and obligately outcrossing, and their high interspecific genetic divergence (mtDNA *cox1* K2P distance 11.2%) [35,36] make this a unique system to investigate epigenetic patterns of hybrids between genetically distant vertebrate species. *K. hermaphroditus* and *K. ocellatus* were sampled from four localities in south and southeast Brazil, two where both species were sympatric (Guaratiba and Fundão; GUA and FUN) and two only inhabited by *K. ocellatus* [35]. Here we only analysed the sympatric (i.e., coexisting) populations. Fish species were identified morphologically and confirmed by cytochrome oxidase subunit I (cox1) barcoding [41]. The nature of the hybrids analysed here was previously described in the first study of hybridization between these two species [41]. Contrary to some theoretical expectations, all F1 hybrids we identified were sired by the rare males of the predominantly selfing species K. *hermaphroditus*, likely because their high selfing rates do not give much opportunity for outcrossing, either intra or interspecific. However, given the difficulties to detangle mating opportunities and hybrid viability in natural populations directly collected in the field, our study does represent a formal test of the evolutionary factors leading to the asymmetrical direction of hybridization.

### Methylation-sensitive genotype-by sequencing library (msGBS) and data processing

A methylation-sensitive genotype-by-sequencing library (msGBS) was initially constructed using pectoral-fin samples of 55 hermaphrodite individuals (33 *K*. ocellatus and 22 *K. hermaphroditus*) as detailed in [41] using a protocol modified from the genotype-by-sequencing protocol described in [42,43]. In brief, genomic DNA was digested using *Eco*RI and *Hpa*II and ligated to barcoded adapters. A single library was produced by pooling 20 ng of DNA from each ligation product and amplified in eight separate PCR reactions that were pooled after amplification, size-selected (range 200–350 bp) and sequenced in an Illumina NextSeq500 sequencer. Paired-end reads were demultiplexed using GBSX v 1.3 [44]. We then filtered (-qtrim r; -minlength 25) and merged the reads by individual using BBmap tools [45] mapped to *Kryptolebias marmoratus* reference genome [46] using Bowtie 2 v. 2.2.3 and generated filtered and indexed individual BAM files with SAMtools v. 1.9 [47]

### Differentially methylated cytosines and hybrid cytosine methylation patterns

To investigate cytosine methylation patterns, we selected 39 individuals with *K. ocellatus* (17) or *K. hermaphroditus* (22) with mtDNA haplotype from the two mangroves where they coexist (GUA and FUN; Supplementary Table S2). Among these, five F1 hybrids and two backcrossess were identified based on 16 microsatellites and SNP data as detailed in Berbel-Filho et al (2021) [41]. Differentially methylated cytosines (DMCs) were identified using the R package msgbsR [48]. Individual restriction-digested reads were aligned to the *K. marmoratus* reference genome [49] and filtered out for correct cut sites and possible outliers. The function *diffMeth* was used to split data according to comparisons, normalize read counts according to library size and identify DMCs. We performed three comparisons: (1) *K. ocellatus* vs *K. hermaphroditus;* (2) hybrids vs *K. ocellatus*; and (3) hybrids vs *K. hermaphroditus*. Only loci with more than one count per million (CPM) reads in at least ‘n’ individuals in each compared group, with ‘n’ being determined by the group with the lowest number of samples in each comparison (11 in *K. ocellatus* vs *K. hermaphroditus*; five in the comparisons including hybrids). DMCs were then filtered with a false discovery rate (FDR) of 0.01 and the logFC value was retrieved to evaluate the intensity and direction of methylation changes. We generated a list of common DMCs (FDR <0.01) present in the comparisons between F1 hybrids vs *K. ocellatus* and F1 hybrids vs *K. hermaphroditus* and the normalized counts of these DMCs across all individuals were used for the downstream analysis.

**Figure 1.**
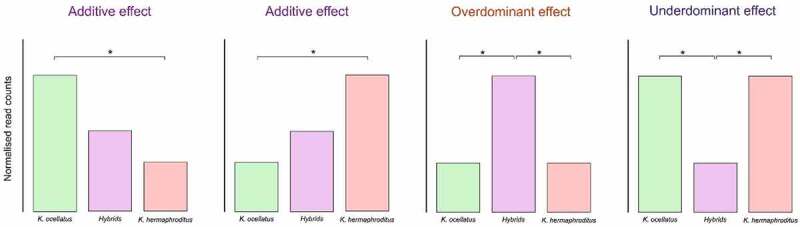
Schematic representation of the classification of differentially methylated cytosines in either potentially additive, overdominant or underdominant in the hybrids compared to the parental species. Asterisks represent significant differential methylation between groups.

To visualize overall variation in DNA methylation, we performed a multidimensional scale analysis (MDS) using Euclidean distance across all individuals using the identified common DMCs. To compare DMC profile across experimental groups using hierarchical clustering, normalized counts per DMC and individual were scaled and the differences in normalized counts for each site were estimated. Inheritance was considered potentially additive if normalized counts of DMCs between the parental species were intermediate in the hybrids and over- or under dominant if normalized counts were higher or lower in hybrids compared to the parental species (Figure 1).

Bray-Curtis genomic (SNP) [41] and epigenomic (methylation) pairwise distances between individuals were estimated using *phyloseq* v1.32.0 [50]. Generalized Linear Mixed-effects models (GLMM) of the pairwise epigenetic distance were fitted using the *lmer* function in *lme4* (Bates, et al. 2014) using genetic pairwise distance, sampling site and species as fixed factors and individual (ID) as random effects. Comparisons between models with and without random factors were carried by AIC comparisons with respect to the GLMM fitted by Maximum Likelihood. The correlation between genetic and epigenetic distances was also analysed with a Mantel test.

### Genomic context and gene ontology enrichment analysis

Using the annotated *K. marmoratus* reference genome [46], we identified the genomic context of the DMCs common to the two comparisons between F1 hybrids and parental species, i.e., within gene body, promoter or intergenic region (Supplementary material). To identify potential differences in DNA methylation across different genomic contexts, we run MDS using DMCs from each group.

The annotated regions affected by these DMCs were used for the gene ontology enrichment analysis using zebrafish (*Danio rerio*) gene orthologs in GOrilla [51]. We searched for enrichment across biological process ontologies curated for zebrafish. Only genes that matched with the gene names annotated for zebrafish were included in the gene ontology analysis.

## Results

### Cytosine methylation patterns in hybrids and parental species

**Figure 2.**
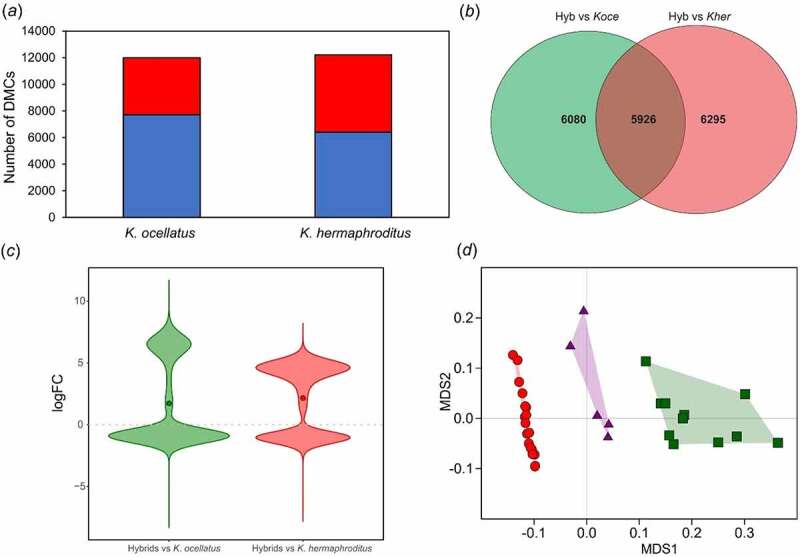
Cytosine methylation comparisons between parental species and F1hybrids. *(a*) Number of differentially methylated cytosines (DMCs) in *K. ocellatus* and *K. hermaphroditus* compared to their hybrids. Hypomethylated (logFC value > 1 in blue) and hypermethylated (logFC value < 1 in red) DMCs in comparison to hybrids are shown in blue and red, respectively. (*b*) Overlap in the number of DMCs of *K. ocellatus* and *K. hermaphroditus* in comparison with their hybrids. (*c*) Direction of changes of the common 5,926 DMCs in the comparisons among hybrids vs parental species. logFC values represent the intensity of changes compared to the hybrid values. logFC > 1 indicates higher CPM read counts (hypomethylation) in the hybrids compared to one of the parental species. logFC < 1 indicates lower CPM read counts (hypermethylation) in the hybrids compared to one of the parental species. Circles represent the mean for each comparison. (*d*) Multidimensional scaling analyses of the normalized counts for the 38,473DMCs between parental species. Green squares represent *K. ocellatus*, red circles represent *K. hermaphroditus*, and triangles represent the F1 hybrids.

The msGBS library of individuals from FUN and GUA (N = 37; 2 backcrosses were excluded from the analyses) yielded in average 6,577,003 reads per individual, with 85.65% reads uniquely mapping to *K. marmoratus* reference genome (ASM164957v1) (Supplementary Tables S1 & S2) corresponding to 830,905 msGBS loci. In total, 37,664 (4.5% of the sequenced loci) significant differentially methylated cytosines (DMCs) were found between *K. ocellatus* and *K. hermaphroditus* (false discovery rate < 0.01). *Kryptolebias hermaphroditus* showed a slightly higher number of DMCs than *K. ocellatus* relative to the hybrids (hybrids vs *K. hermaphroditus*: 12,221 DMCs (1.5% of the sequenced loci); hybrids vs *K. ocellatus*: 12,006 DMCs (1.4% of the sequenced loci)) (Figure 2a), with 47.51% of the DMCs hypermethylated in *K. hermaphroditus* in relation to the hybrids (and *vice versa* for hypomethylated DMCs) compared to 35.69% in *K. ocellatus* (Figure 2a). MDS analysis using DMCs between parental species positioned the hybrids between two clusters representing the parental species (Figure 2d). These results were also supported by the MDS using all reads normalized by library size (830,905 sites) (Supplementary Figure S1). In both cases, individuals identified as F1 hybrids occupied eigenspaces in between the parental species.

Most F1 hybrids displayed intermediate levels of DNA methylation relative to the parental species in the hierarchical clustering analysis (Supplementary Figure S2), suggesting a genetic component underlying the methylation variability. Of the 37,664 DMCs between *K. ocellatus* and *K. hermaphroditus*, 31,145 (82.7%) had intermediate normalized read counts in the hybrids compared to the parental species. The remaining 6,619 (17.3%) DMCs had either higher (55.3%) or lower (44.7%) number of normalized reads in hybrids when compared to parental species. Hybrids shared 5,926 common DMCs with both parental species (Figure 2b), of which 39.39% and 60.61% were hyper and hypo methylated, respectively, in *K. ocellatus* in relation to the hybrids (59.97.% and 40.03% in *K. hermaphroditus*; Figure 2c). The same general pattern was observed in the analysis of the 5,926 DMCs common in the comparisons between hybrids parental species, with 97.8% showing intermediate number of reads in hybrids, while 2.2% were either over or under dominant in hybrids (77 and 54 DMCs, respectively).

The comparison between two models including and excluding individual (ID) as random factor, indicated that the model that included pairwise genetic distance, species and ID provided the best fit to the data (lmer(Methylation ~ SNPs + SamplingSite1 + SamplingSite2 + Species1 + Species2 + (1|c1) + (1|c2)), based on AIC and model comparison by likelihood ratio test (Supplementary material Table S3). Mantel tests based on Pearson’s product-moment correlation indicated that pairwise epigenomic and genomic distances were weakly but significantly correlated when all the individuals were considered (r = 0.157, P = 0.006) but not when the species were analysed separately (Kher: r = 0.141 P = 0.817; Koce: r = – 0.078 P = 0.738; F1 hybrids r = 0.133 P = 0.375).

### Gene ontology analysis

Of the 5,926 DMCs shared between hybrids and parental species, 264 (4.45%) were within 2kb upstream gene bodies, representing putative promoters, 3,671 (61.95%) were overlapping gene bodies, while 783 (13.21%) represented potential intergenic regions. Of these, 1,208 (20.38%) were within unannotated regions and the rest affected putative promoters and/or gene bodies of 3,043 putative unique genes, 1,677 of them mapping to orthologs in the zebrafish genome (1550 genes associated to a GO term). The gene ontology enrichment analysis identified significantly overrepresented ontologies influencing 63 biological processes, 39 molecular functions, and 10 cellular components (Supplementary File 2). The MDS analysis separating DMCs by genomic context revealed a clear clustering between parental species across all genomic contexts (e.g., promoters, gene bodies, and intergenic regions), with the hybrids forming an intermediate cluster differentiated from the parental species. The variance in the methylation of the promoters was very low in the case of the selfing *K. hermaprhoditus* but higher for the other groups. (Supplementary material Figure S3).

## Discussion

The hybrids between two mangrove killifish species with different mating systems, *K. hermaphroditus* (predominantly selfing) and *K. ocellatus* (obligately outcrossing) displayed differentially methylated cytosines with respect to both parental species, suggesting that hybridization may play a role in increasing their epigenetic variation. The epigenetic inheritance patterns of the hybrids were predominantly additive, with intermediate levels of cytosine methylation relative to the parental species, which likely reflects the strong influence of the genetic background on cytosine methylation [40,52]. However, we also found a small proportion of DMCs with non-additive, over- or under-dominant effects in the hybrids. Hybridization between closely related self-fertilizing *Kryptolebias* lineages (3% K2P distance at *cox1*, compared to 11% here) had been previously observed [53], potentially representing a source of novel genetic and epigenetic variation. In the selfing and predominantly inbred mangrove killifish species *K. marmoratus* and *K. hermaphroditus*, genetic diversity is also known to increase by occasional male-mediated outcrossing [40,54]. Given that *K. marmoratus* males prefer to associate with genetically dissimilar hermaphrodites [55] and that increased heterozygosity is related to lower parasite loads [40,54], it is possible that mechanisms that increase genetic diversity are important for the fitness and survival of the species. In addition, as for other asexual and selfing lineages, including asexually reproducing hybrid fish, epigenetic-mediated phenotypic plasticity, and inheritance can represent important sources of variation to respond to environmental challenge [33,56].

Reproductive isolation tends to increase with genetic distance between species pairs [57] potentially due to postzygotic hybrid incompatibility caused by the gradual accumulation of divergent alleles. *Kryptolebias ocellatus* and *K. hermaphroditus* are very divergent genetically [58,59] and, as we have shown here, also epigenetically, yet at least some of their hybrids are viable. Previous evidence of a close relationship between the genome and methylome in response to environmental variation in mangrove killifishes [60,61], and the observed relationship between genomic and epigenomic pairwise distances, suggest that the DNA methylation differences between *K. ocellatus* and *K. hermaphroditus* could be primarily driven by genomic differences between the species. Thus, while we cannot rule out that microecological differences between the species (e.g., diet, habitat use) may have influenced their DNA methylation profiles, the intermediate nature of the methylation patterns found between *K. ocellatus* and *K. hermaphroditus* hybrids highlight the importance of genetic background on DNA methylation levels, also found in F1 hybrids of other fish systems [14]. Yet, a small proportion of the DMCs between hybrids and the parental species displayed non-additive effects in the hybrids. Most studies in plants using methylation-sensitive amplification fragment length polymorphisms (MS-AFLP) indicate prominent additive effects in cytosine methylation patterns, with typical Mendelian inheritance [26,27] with only few examples of non-additive effects in hybrids, potentially related to phenotypic plasticity and adaptation [62]. Although few studies have assessed DNA methylation inheritance in fish hybrids [14], the majority of DNA methylation effects appear to be additive, at least in allopolyploid hybrids [63], as observed here. This contrasts with transgressive patterns of gene expression found in some F1 fish hybrids [18,21,64], but generally agree with patterns found in hybrids among more genetically distant species such as *Drosophila* species and the house mouse, where divergent traits are regulated by more genes without a dominance pattern [22,23]. However, high-throughput sequencing has further revealed the importance of non-additive DNA methylation states in crop hybrids [28,29] which could alter gene expression levels [65]. Thus, although most genes are additively expressed in hybrids, there are thousands of changes in transcript levels that are non-additive, some of them related to epigenetic modifications, likely to be involved in hybrid heterosis or incompatibility [66]. These non-additive epigenetic components could represent stochastic epimutations that would generate phenotypic diversity [25], and potentially increase hybrid fitness [5]. Compared to plants, there is not much information in animals on the effects of methylation patterns on hybrid fitness. It has been suggested that DNA methylation could be related to higher hybrid performance or heterosis in farmed fish but the underlying mechanisms are unclear [67]. In plants, such as rice or *Arabidopsis*, non-additive methylation has been mainly observed in loci that are differentially methylated in the parental species, and it has been suggested that these could contribute to heterosis or hybrid vigour, trough changes in gene expression related to hybrids development or functionality [68]. In fact, non-additive changes in gene expression potentially related to heterosis have been associated with differentially methylated genes in hybrids [69]. At least part of the non-additive methylation in hybrids could correspond to stochastic epigenetic mutations [70]. These stochastic epimutations, if associated with adaptive phenotypes, could contribute to the survival of populations with low genetic diversity in changing environments, at least in the short term and potentially in the long term if they were inherited [71], and can play an important role in the survival of the hybrids.

Based on the gene ontology results, the DMCs found between hybrids and both parental species may be expected to affect important biological processes, many of them involved in developmental processes (i.e., central nervous system development, chordate embryonic development, eye development). As at least some of the hybrids are viable and fertile (based on backcrosses found in [41]), these DMCs do not seem detrimental and could reflect allele-specific compensatory effects, with stabilizing selection favouring an optimal level of gene expression by compensating the effects of single alleles through *cis* and *trans* regulatory factors [72]. However, gene expression analyses (not possible here due to the type of preservation of the samples) are necessary to assess the existence of compensatory DNA methylation inheritance and epiallele dominance [28].

In summary, we found predominantly additive epigenetic effects (intermediate methylation levels) in the hybrids between two genetically divergent fish with different mating systems. However, a small percentage of non-additive effects was also detected, which was common to both parental species and their hybrids and which was unlikely to have been related to environmental effects. Whether such non-additive epigenetic variation represents an evolutionary bet-hedging strategy for asexual or self-fertilizing species living in rapidly changing environments is not clear and merits further research. In addition, as for plants, understanding how epigenetic modifications in hybrids alter physiological and metabolic gene networks could be used to improve performance in animal breeding, by exploiting potential heterosis effects.

## Supplementary Material

Supplemental Material

## Data Availability

FastaQC files for GBS library can be accessed at NCBI (accession PRJNA563625). Sequence and microsatellite data are available in Supplementary Material of Berbel-Filho et al. (2021)^41^. https://github.com/waldirmbf/BerbelFilho_etal_KryptolebiasHybridisation/tree/master/1.ProcessingSequencingFiles/1.2.EpigeneticAnalysis.
