# Supplementary material for: Additive and non-additive epigenetic signatures of natural hybridization between fish species with different mating systems
Source: Epigenetics. 2022 Sep 15;17(13):2356–65. doi: 10.1080/15592294.2022.2123014 (PMC9665120; doi:10.1080/15592294.2022.2123014)
Supplement: Supplemental Material [file KEPI_A_2123014_SM7941.docx]

**Supplementary Material**

**Additive and non-additive epigenetic signatures of natural hybridisation between fish species with different mating systems**

Running head: Epigenetics of fish hybrids

Waldir M. Berbel-Filho^1§^, George Pacheco^2^, Mateus G. Lira^3^, Carlos Garcia de Leaniz^1^, Sergio M. Q. Lima^3^, Carlos M. Rodríguez-López^4^, Jia Zhou^5^ and Sofia Consuegra^1*^

^1^ Centre for Sustainable Aquatic Research, Department of Biosciences, College of Science, Swansea University, Swansea, UK.

^2^ Section for Evolutionary Genomics, The Globe Institute, Faculty of Health and Medical Sciences, University of Copenhagen, 1353 Copenhagen, Denmark.

^3^ Laboratório de Ictiologia Sistemática e Evolutiva, Departamento de Botânica e Zoologia, Universidade Federal do Rio Grande, Natal, Brazil.

^4^ Environmental Epigenetics and Genetics Group, Department of Horticulture, College of Agriculture, Food and Environment, University of Kentucky, Lexington, KY, USA.

^5^ State Key Laboratory of Plant Genomics, Institute of Microbiology, Chinese Academy of Sciences, Beijing, China

*Corresponding author: [s.consuegra@swansea.ac.uk](mailto:s.consuegra@swansea.ac.uk)

^§^Current address: Department of Biology, University of Oklahoma, Norman, OK, USA

**Supplementary Figure S1**. Multidimensional scaling analysis (MDS) of the normalised counts for all 830,950 sites with reads in the msGBS library Green squares represent *K*. *ocellatus,*  red circles represent *K*. *hermaphroditus,* and purple triangle represent F1 hybrids.


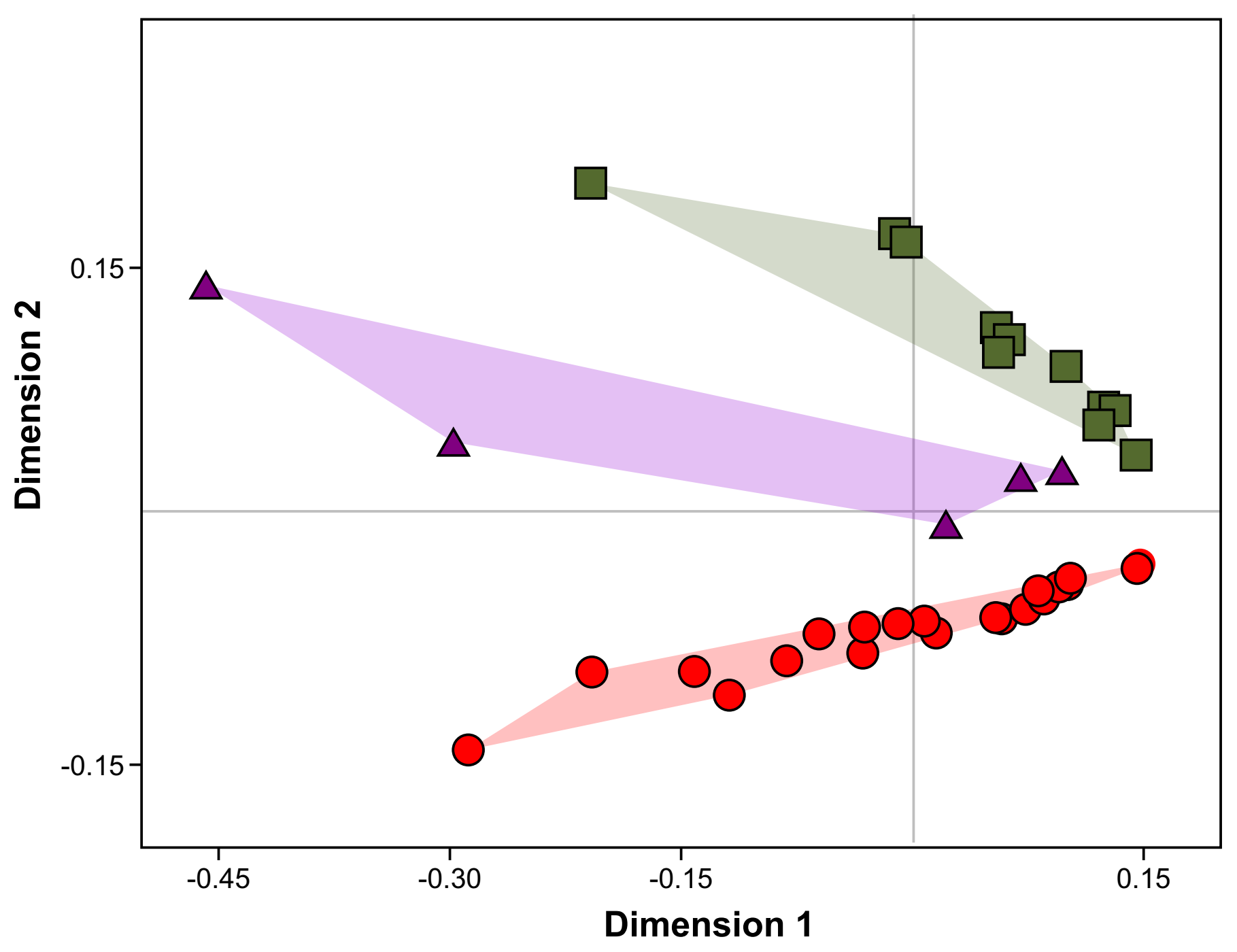


**Supplementary Figure S2.** Hierarchical clustering of the scaled normalised counts for the 5,92600 differentially methylated cytosines (DMCs) common to both comparisons between hybrids and parental species. Each cell represents an individual DMC, and each column represent an individual fish. Hypomethylated DMCs in red and hypermethylated DMCs in blue.


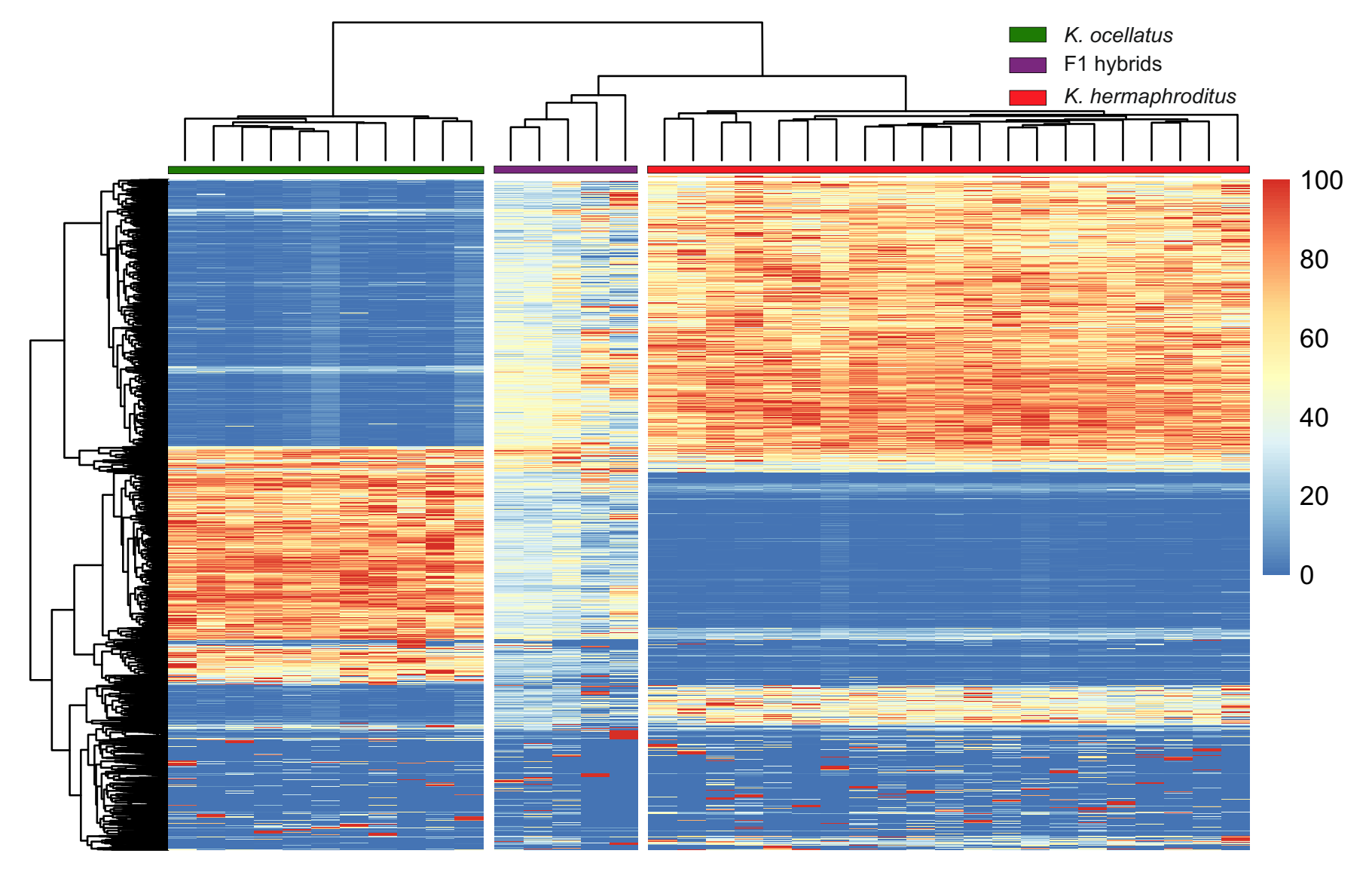


**Supplementary Figure S3**. Multidimensional scaling analysis (MDS) of the normalised counts for the 5,926 DMCs common to the comparisons between hybrids and parental species in (*a*) putative promoters (within 2kb upstream the transcription start site), (*b*) gene bodies (*c*) intergenic regions (≥2kb upstream of TSS or downstream the gene bodies. Squares for *K*. *ocellatus,* circles for *K*. *hermaphroditus,* and triangle for F1 hybrids.


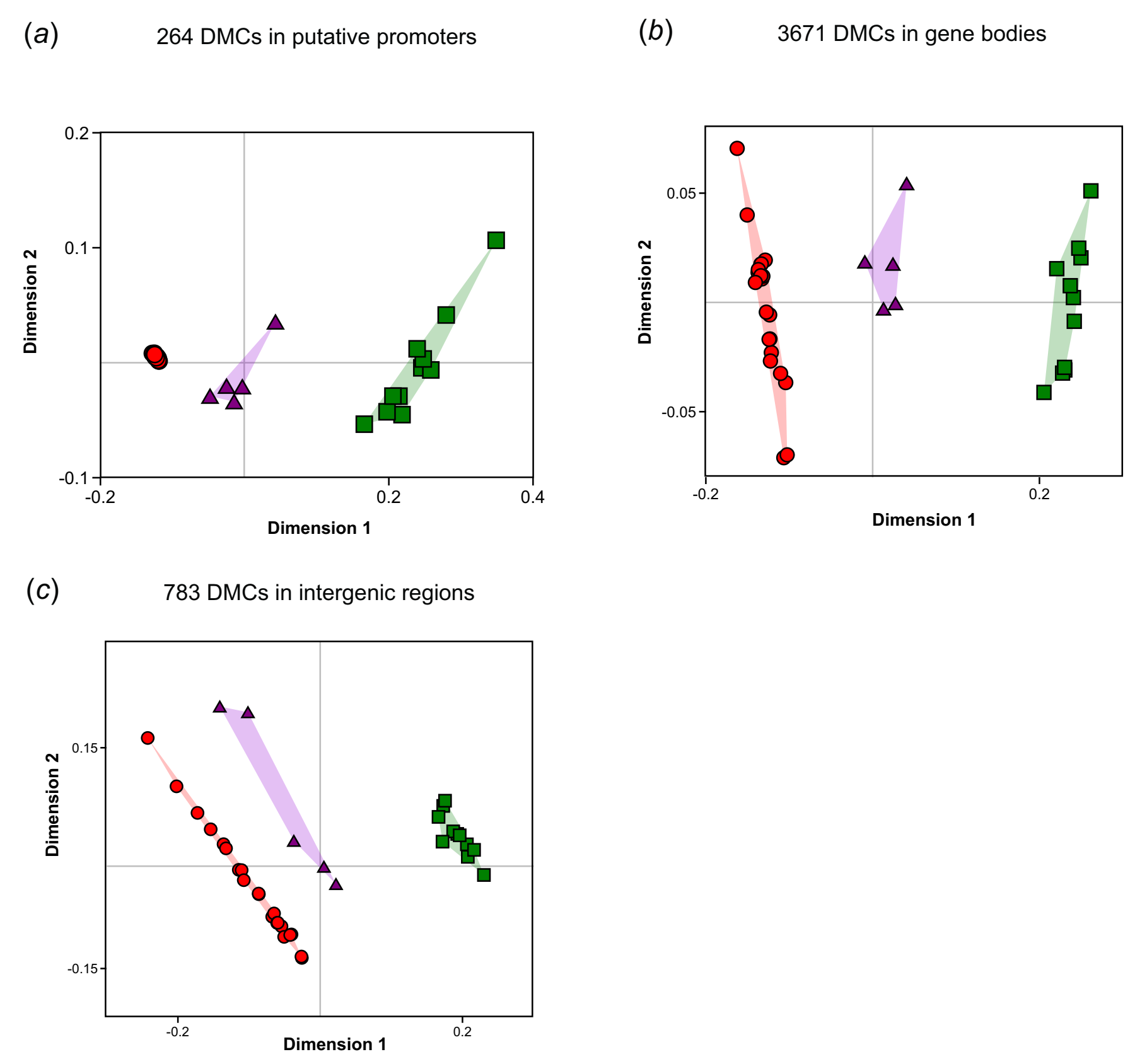


**Supplementary Table S1**. Individuals with *Kryptolebias ocellatus* and *Kryptolebias hermaphroditus,* classified according *cox1* haplotypes in Berbel-Filho*, et al.* (2020)^1^, used for the epigenetic analyses with sampling locations and their respective sampling sizes for microsatellites, single-polymorphism analysis (SNPs) and methylation-sensitive genotype-by-sequencing (msGBS) library. ‘Reference for Msats’ refer to references from which microsatellite data was extracted. RJ, Rio de Janeiro State. Two backcrosses, one with *Kryptolebias ocellatus* and one with *Kryptolebias hermaphroditus* mtDNA were excluded from the analyses.

| **Sample ID** | **Location** | **Latitude** | **Longitude** | **Msats** | **SNPs** | **msGBS** | **Reference for Msats** |
| --- | --- | --- | --- | --- | --- | --- | --- |
| (a) *Kryptolebias ocellatus* | | | | | | | |
| FUN | Fundão mangrove, Rio de Janeiro, RJ | 22°52'2.50"S | 43°13'27.50"W | 11 | 6 | 6 | Berbel-Filho et al. (2020) |
| GUA 2017 | Piracão mangrove, Guaratiba, RJ | 23° 0'1.90"S | 43°34'51.50"W | 19 | 11 | 11 | Berbel-Filho et al. (2020) |
| **Total** |  |  |  | **30** | **17** | **17** |  |
| (b) *Kryptolebias hermaphroditus* | | | | | | | |
| FUN | Fundão mangrove, Rio de Janeiro, RJ | 22°52'2.50"S | 43°13'27.50"W | 16 | 10 | 10 | Berbel-Filho et al. (2020) |
| GUA 2017 | Piracão mangrove, Guaratiba, RJ | 23° 0’1.90”S | 43°34’51.50”W | 16 | 12 | 12 | Berbel-Filho et al. (2020) |
| **Total** |  |  |  | **32** | **22** | **21** |  |

**Supplementary Table S2**. Summary of 37 samples sequenced in the methylation-sensitive genotype-by-sequencing (msGBS) library. F1 hybrids marked with asterisks.

| **ID** | **Species**  **(identified using *cox1*)** | **Sampling location** | **Number of reads** | **Uniquely mapped (%)** |
| --- | --- | --- | --- | --- |
| GUA_27 | *K. hermaphroditus* | GUA | 8,962,202 | 90.30 |
| GUA_28 | *K. hermaphroditus* | GUA | 4,161,657 | 90.87 |
| GUA_29 | *K. hermaphroditus* | GUA | 13,831,746 | 90.29 |
| GUA_30 | *K. hermaphroditus* | GUA | 7,000,238 | 90.05 |
| GUA_31 | *K. hermaphroditus* | GUA | 6,646,432 | 89.95 |
| GUA_37 | *K. hermaphroditus* | GUA | 3,013,973 | 90.06 |
| GUA_39 | *K. hermaphroditus* | GUA | 3,472,656 | 89.73 |
| GUA_40 | *K. hermaphroditus* | GUA | 13,651,662 | 90.50 |
| GUA_41 | *K. hermaphroditus* | GUA | 5,348,736 | 90.08 |
| GUA_42 | *K. hermaphroditus* | GUA | 1,786,528 | 91.00 |
| GUA_43 | *K. hermaphroditus* | GUA | 7,269,128 | 89.39 |
| GUA_44 | *K. hermaphroditus* | GUA | 7,937,202 | 89.92 |
| FUN_20 | *K. hermaphroditus* | FUN | 8,493,250 | 89.83 |
| FUN_21 | *K. hermaphroditus* | FUN | 4,140,936 | 90.70 |
| FUN_22 | *K. hermaphroditus* | FUN | 4,990,777 | 90.96 |
| FUN_24 | *K. hermaphroditus* | FUN | 3,520,363 | 89.86 |
| FUN_25 | *K. hermaphroditus* | FUN | 4,421,739 | 90.34 |
| FUN_29 | *K. hermaphroditus* | FUN | 7,611,704 | 89.30 |
| FUN_30 | *K. hermaphroditus* | FUN | 2,892,977 | 88.63 |
| FUN_31 | *K. hermaphroditus* | FUN | 9,160,363 | 89.53 |
| FUN_32 | *K. hermaphroditus* | FUN | 2,322,888 | 88.84 |
| GUA_04 | *K. ocellatus* | GUA | 5,033,372 | 77.40 |
| GUA_05 | *K. ocellatus* | GUA | 11,979935 | 75.68 |
| GUA_06 | *K. ocellatus* | GUA | 6,418,126 | 81.00 |
| GUA_10 | *K. ocellatus* | GUA | 11,097,202 | 81.18 |
| GUA_11 | *K. ocellatus* | GUA | 2,208,760 | 75.05 |
| GUA_13 | *K. ocellatus* | GUA | 11,446,137 | 77.19 |
| GUA_16 | *K. ocellatus* | GUA | 14,282,577 | 80.18 |
| GUA_17* | *K. ocellatus* | GUA | 4,522,779 | 72.17 |
| GUA_18 | *K. ocellatus* | GUA | 7,266,866 | 79.34 |
| GUA_19 | *K. ocellatus* | GUA | 8,150,733 | 81.55 |
| FUN_06 | *K. ocellatus* | FUN | 5,098,517 | 81.78 |
| FUN_08* | *K. ocellatus* | FUN | 9,433,950 | 86.75 |
| FUN_10 | *K. ocellatus* | FUN | 6,856,002 | 81.10 |
| FUN_11* | *K. ocellatus* | FUN | 7,773,860 | 86.68 |
| FUN_43* | *K. ocellatus* | FUN | 578,575 | 83.31 |
| FUN_47* | *K. ocellatus* | FUN | 564,546 | 78.82 |
| **Average** |  |  | **6,577,003** | **85.65** |

**Supplementary Table S3**. Generalised Linear Mixed-effects models (GLMM) of the influence of genetic pairwise distance, sampling site and species on epigenetic pairwise distance with individual (ID) as random effects as well as model comparisons with and without random factors.

#Mod1 <- lmer((Methylation) ~ SNPs + SamplingSite1 + SamplingSite2 + Species1 + Species2 + (1|c1) + (1|c2), GLMM_metadata)

#Mod2 <- lm(GLMM_metadata$Methylation ~ SNPs + SamplingSite1 + SamplingSite2 + Species1 + Species2, GLMM_metadata)

> anova(Mod1,Mod2)

refitting model(s) with ML (instead of REML)

Data: GLMM_metadata

Models:

Mod2: GLMM_metadata$Methylation ~ SNPs + SamplingSite1 + SamplingSite2 + Species1 + Species2

Mod1: (Methylation) ~ SNPs + SamplingSite1 + SamplingSite2 + Species1 + Species2 + (1 | c1) + (1 | c2)

npar AIC BIC logLik deviance Chisq Df Pr(>Chisq)

Mod2 9 -3870.0 -3829.5 1944.0 -3888.0

Mod1 11 -4613.1 -4563.6 2317.6 -4635.1 747.12 2 < 2.2e-16 ***

> summary(Mod1)

Random effects:

Groups Name Variance Std.Dev.

c1 (Intercept) 8.741e-05 0.009349

c2 (Intercept) 3.117e-05 0.005583

Residual 4.092e-05 0.006397

Number of obs: 666, groups: c1, 36; c2, 36

Fixed effects:

Estimate Std. Error df t value Pr(>|t|)

(Intercept) 0.082207 0.005546 66.158388 14.823 < 2e-16 ***

SNPs 0.006061 0.000885 638.798009 6.849 1.75e-11 ***

SamplingSite1GUA -0.001586 0.003692 34.099839 -0.430 0.67016

SamplingSite2GUA 0.002097 0.002242 21.572946 0.936 0.35982

Species1Kher -0.015745 0.004905 32.230663 -3.210 0.00300 **

Species1Koce -0.029630 0.005940 35.447623 -4.989 1.61e-05 ***

Species2Kher -0.024185 0.004057 40.086545 -5.961 5.33e-07 ***

Species2Koce -0.014376 0.004485 37.272463 -3.206 0.00276 **

Signif. codes: 0 '***' 0.001 '**' 0.01 '*' 0.05 '.' 0.1 ' ' 1

**References**

1. Berbel-Filho WM, Tatarenkov A, Espírito-Santo HMV, Lira MG, Garcia de Leaniz C, Lima SMQ, Consuegra S. More than meets the eye: syntopic and morphologically similar mangrove killifish species show different mating systems and patterns of genetic structure along the Brazilian coast. Heredity 2020; 125:340-52.
